# Diabetes Preventive Care Practices in North Carolina, 2000–2015

**DOI:** 10.5888/pcd15.170316

**Published:** 2018-03-22

**Authors:** Huabin Luo, Ronny A. Bell, Doyle M. Cummings, Zhuo (Adam) Chen

**Affiliations:** 1 East Carolina University, Greenville, North Carolina; 2 University of Georgia, Athens, Georgia; 3 University of Nottingham, Ningbo, China

## Abstract

This analysis assessed trends in measures of diabetes preventive care overall and by race/ethnicity and socioeconomic status in the North Carolina Behavioral Risk Factor Surveillance System (2000–2015). We found increasing trends in 5 measures: diabetes self-management education (DSME), daily blood glucose self-monitoring, hemoglobin A_1c_ tests, foot examinations, and flu shots. Non-Hispanic black and non-Hispanic white respondents showed increases in blood glucose self-monitoring, and a significant time-by-race interaction was observed for annual flu shots. Predisposing, enabling, and need factors were significantly associated with most measures. DSME was positively associated with 7 measures. Expanding access to health insurance and health care providers is key to improving diabetes management, with DSME being the gateway to optimal care.

## Objective

North Carolina is one of 15 southern states identified as being in the “diabetes belt” by the Centers for Disease Control and Prevention (1). The state has the 13th highest prevalence of diabetes in the United States. Racial/ethnic disparities in diabetes prevalence are also significant in North Carolina; in 2013, 11.0% of non-Hispanic white adults had diabetes, compared with 15.4% of non-Hispanic black adults (2). This study assessed recent trends in receipt of diabetes preventive care among adults who received a diagnosis of diabetes in North Carolina and whether the gap by race/ethnicity and by socioeconomic status narrowed.

## Methods

Data were from the Behavioral Risk Factor Surveillance System (BRFSS) in 2000, 2002–2010, 2012, 2013, and 2015, years in which North Carolina administered the diabetes module (3). The sample included 17,847 adults (≥18 y) with diagnosed diabetes. BRFSS respondents were asked whether they had been told by a doctor that they had diabetes and whether they participated in 4 self-care activities (engaging in any physical activity or exercise in the past month, daily blood glucose self-monitoring, checking feet daily for sores or irritations, and participating in diabetes self-management education [DSME]) and 4 clinical care services (≥2 hemoglobin A_1c _[HbA_1c_] tests in the past 12 months, annual dilated eye examination, annual foot examination by a health professional, and annual flu shots) (4).

Covariates were chosen according to Andersen’s model of health service utilization (5). Predisposing factors were age, sex, race/ethnicity (non-Hispanic white, non-Hispanic black, and other [data on Hispanic respondents and other racial groups were combined because of small sample sizes]), and marital status. Enabling factors were annual household income, educational attainment, employment status, health insurance coverage, and having a regular health care provider. Need factors were taking insulin (an indicator of diabetes severity) and self-rated general health status.

We first calculated the weighted rates of receipt of the 8 diabetes preventive care items and assessed the temporal linear trends of the rates with the survey year as an independent variable. Second, controlling for age, sex, and marital status, we calculated the predicted margins of the 8 measures among non-Hispanic white and non-Hispanic black respondents and compared the rate of change for these 8 measures. Third, we ran multivariate logistic regression models for the 8 measures and tested a time-by-race interaction and a time-by-socioeconomic status interaction (ie, time × income, and time × education). All analyses were conducted by using the Stata SVY routine to account for survey design (3).

## Results

**Figure 1:**
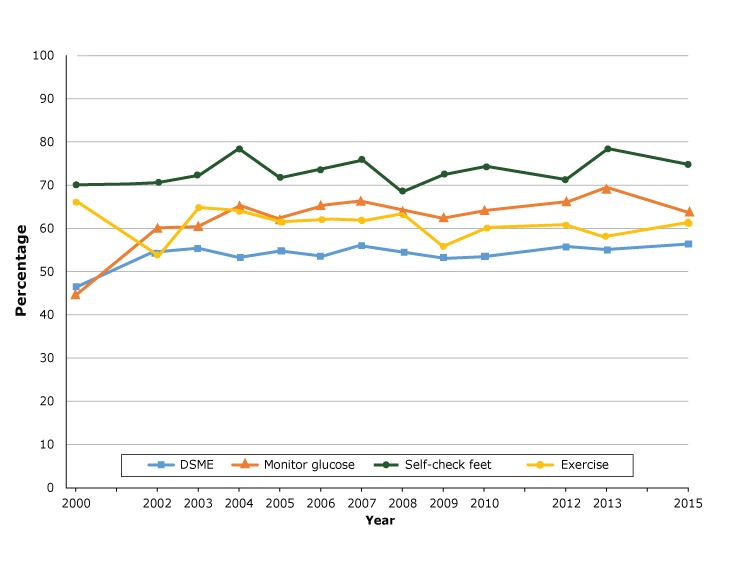
Proportion of adults with diabetes that participated in self-care activities in North Carolina, Behavioral Risk Factor Surveillance System, 2000–2015. Abbreviation: DSME, diabetes self-management education. **Table:** 

**Figure 2:**
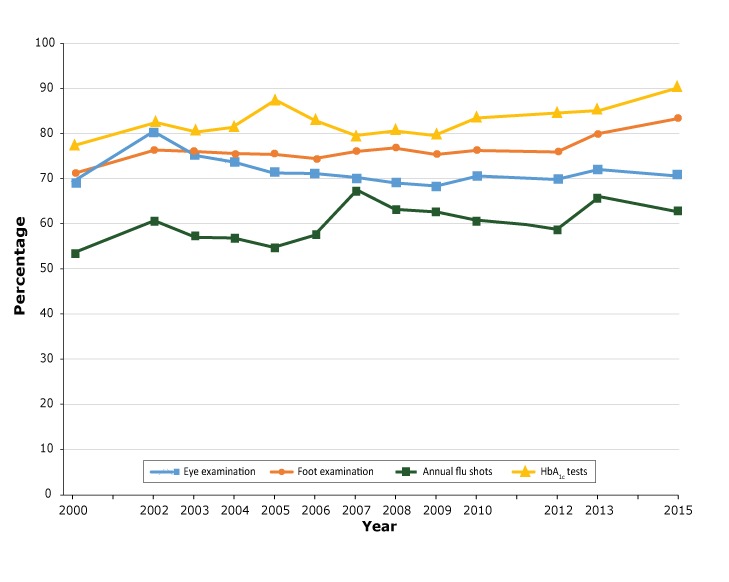
Proportion of adults with diabetes that received clinical care services in North Carolina, Behavioral Risk Factor Surveillance System, 2000–2015. **Table:** 

From 2000 to 2015, rates of DSME and daily blood glucose self-monitoring increased significantly, from 46.5% to 56.4% (*P* = .03), and from 44.7% to 64.5% (*P* = .01), respectively (Figure 1). During the same period, rates of annual foot examination by a health professional, annual flu shots, and at least 2 HbA_1c_ tests in past 12 months increased significantly, from 71.6% to 83.7% (*P* < .001), 53.4% to 63.1% (*P* = .03), and 78.2% to 93.1% (*P* = .02), respectively (Figure 2).

Year	DSME, %	Daily Blood Glucose Monitoring, %	Checking Feet Daily, %	Exercise, %
2000	46.5	44.7	70.1	66.0
2002	54.0	60.3	70.8	53.2
2003	55.5	60.2	72.3	64.7
2004	53.1	65.3	78.3	64.1
2005	54.8	62.2	71.8	61.5
2006	53.8	65.2	73.5	62.1
2007	56.0	66.7	76.1	61.7
2008	54.6	64.0	68.5	63.5
2009	53.5	62.8	72.4	55.8
2010	53.7	64.0	74.1	59.7
2012	55.8	66.1	71.2	60.1
2013	55.5	69.7	78.0	58.3
2015	56.4	64.5	74.8	61.3

Year	Annual Eye Examination, %	Annual Foot Examination %,	Annual Flu Shot, %	≥2 HbA1c Tests in Past 12 Months, %
2000	69.6	71.6	53.4	78.2
2002	80.5	76.3	60.7	83.0
2003	75.4	76.4	57.5	80.9
2004	74.0	75.5	57.2	81.8
2005	71.6	75.6	54.7	87.8
2006	71.3	74.7	57.6	83.2
2007	70.5	76.3	67.7	79.9
2008	69.6	77.1	63.6	80.9
2009	68.5	75.7	62.8	80.2
2010	70.9	76.7	60.9	83.9
2012	70.1	76.3	59.1	85.0
2013	72.2	80.2	66.0	85.4
2015	71.2	83.7	63.1	91.1

Over time, we found a significant change in rates of daily blood glucose self-monitoring among both non-Hispanic white and non-Hispanic black respondents, with an average annual increase of 0.6% and 1.4%, respectively. We found significant changes in rates of foot examinations by a provider (an average annual increase of 0.7%) and HbA_1c_ tests (an annual increase of 0.5%) only among non-Hispanic white respondents and a significant change in rates of flu shots (an average annual increase of 1.6%) only among non-Hispanic black respondents.

Most of the predisposing, enabling, and need factors were significantly associated with the 8 diabetes preventive care measures. Income, insurance, and education were significant for all 8 measures except for self-checking feet and foot examinations by a provider; a regular provider was significant for all 8 measures except for doing exercise. DSME was positively associated with all other 7 measures. 

The 3 interaction terms (time × race, time × income, and time × education) were not significant, except that 1) the time-by-college interaction in the blood glucose self-monitoring model indicated that adults with some college education were significantly less likely to check blood glucose from 2010 to 2015 than in earlier years in our study; and 2) the time-by-non-Hispanic-black interaction in the model for annual flu shots indicated that non-Hispanic black respondents made more progress than non-Hispanic white respondents in receiving annual flu shots, but they were still less likely to receive flu shots than non-Hispanic white respondents.

## Discussion

We showed an increasing trend in 5 of 8 measures of diabetes preventive care among adults in North Carolina from 2000 to 2015: DSME, blood glucose self-monitoring, HbA_1c_ tests, foot examinations by a health professional, and annual flu shots. We found no consistent patterns of disparities between non-Hispanic black and non-Hispanic white adults. Similar results were reported in other states — non-Hispanic black adults were significantly less likely than non-Hispanic white adults to report having HbA_1c_ tests but more likely to report receiving foot examinations (6). Nonetheless, our study results should be interpreted with caution because preventive care practices may not necessarily translate into better outcomes (7). The time-by-race interaction showed that gaps in rates of annual flu shots between races narrowed. Non-Hispanic black adults had larger gains than non-Hispanic white adults in self-reported receipt of annual flu shots from 2000 to 2015. However, the self-reported flu vaccination rate of non-Hispanic black adults lagged behind that of non-Hispanic white adults.

DSME is a consistent significant contributing factor in other 7 diabetes care measures. However, only 56.4% of adults in our study reported participating in DSME in 2015, lower than the Healthy People 2020 target of 62.5% (8). DSME has been shown to control diabetes complications and to reduce hospital admissions and health care costs (9). To increase participation in and availability of DSME, key barriers, such as lack of or insufficient reimbursement and a mandate for provider referrals to DSME, should be addressed (10).

The most consistent enabling factors of diabetes preventive care were a regular provider, health insurance coverage, and education level. Income was negatively associated with daily blood glucose self-monitoring, and the time-by-income interaction was significant. Individuals with higher income may focus more on other types of self-care than on blood glucose self-monitoring. No consensus exists on whether all patients should monitor their blood glucose, especially nonusers of insulin (11). Future research is needed to assess the effects of blood glucose monitoring on patient satisfaction and health-related quality of life (12). In summary, expanding health insurance and access to a regular provider are key to improving diabetes preventive care, with DSME being the gateway to optimal diabetes preventive care.
